# The Concentration of Potentially Toxic Elements (PTEs) in Indonesian Rice and Their Health Risk Assessment

**DOI:** 10.3390/foods14213652

**Published:** 2025-10-26

**Authors:** Fitria Yuliani, Didah Nur Faridah, Nuri Andarwulan, Dominika Średnicka-Tober

**Affiliations:** 1 Center for Plant Product Quality Testing, Ministry of Agriculture of the Republic of Indonesia, Jl. AUP Pasar Minggu, Jakarta Selatan 12520, Indonesia; v3ayuliani@apps.ipb.ac.id; 2 Department of Food Science and Technology, Faculty of Agricultural Technology, IPB University, IPB Dramaga Campus, Bogor 16680, West Java, Indonesia; didah_nf@apps.ipb.ac.id; 3 Southeast Asian Food and Agricultural Science and Technology (SEAFAST) Center, IPB University, IPB Dramaga Campus, Bogor 16680, West Java, Indonesia; 4 Department of Functional and Organic Food, Institute of Human Nutrition Sciences, Warsaw University of Life Sciences, Nowoursynowska 159c, 02-776 Warsaw, Poland

**Keywords:** rice, exposure assessment, heavy metals, risk assessment

## Abstract

This study assessed the exposure to Potentially Toxic Elements (PTEs) Cd, Pb, As, and iAs in rice collected from 30 provinces in Indonesia using data from the national Total Diet Study (TDS). As rice is the main staple food and a major source of contaminant intake, the study estimated dietary exposure and evaluated the associated health risks across different age groups. The objective was to estimate exposure to PTEs (Cd, Pb, As and iAs) through rice consumption and to evaluate their associated health risks across various age groups. Cd concentrations ranged from 0 to 136 µg/kg, Pb from 0 to 684 µg/kg, total As from 0 to 609 µg/kg, and iAs from 0 to 267 µg/kg. On average, all levels were below the maximum limits set by Codex Alimentarius Standard 193-1995 (2023 revision). Cooking reduced Cd content by 76% and increased Pb levels by 1403%, while As levels remained unchanged. Estimated daily exposures reached 0.134–0.248 µg/kg bw/day for Cd, 0.258–0.475 µg/kg bw/day for Pb, 0.348–0.642 µg/kg bw/day for total As, and 0.236–0.435 µg/kg bw/day for iAs. Exposure to Cd posed a minimal health risk, remaining below the Provisional Tolerable Monthly Intake (PTMI). In contrast, Pb exposure showed a Margin of Exposure (MOE) greater than 1, suggesting a low level of concern as defined by the EFSA CONTAM Panel, while iAs exposure resulted in an MOE below 10,000, indicating a possible health risk. Pb exposure from cooked rice consumption may pose a potential risk of IQ reductions of up to 2.59 ± 0.95 points in toddlers and 5.27 ± 1.93 points in children. These findings highlight the need to implement food safety risk management strategies in rice production and processing, particularly given the high rice consumption in Indonesia.

## 1. Introduction

Food safety has become a major concern worldwide. The consumption of food contaminated with potentially toxic elements (PTEs) is a worldwide concern due to their harmful impact on human health. Elements such as arsenic (As), aluminum (Al), barium (Ba), cadmium (Cd), chromium (Cr), lead (Pb), and titanium (Ti) are considered non-essential and potentially toxic. These elements can accumulate in rice grown in contaminated soil and water. As a result, rice consumption may substantially contribute to health risks related to exposure. In recent times, cumulative exposure to multiple contaminants has become a crucial concern in the field of food safety [1,2].

Food contamination by heavy metals has been regarded as a serious issue due to their stable, non-degradable, and persistent nature. Human exposure to heavy metals can occur through three main pathways: ingestion, inhalation, and skin contact; however, the most significant exposure to heavy metals in humans is food consumption. Heavy metal exposure can damage organs even at low levels. These metals (such as Pb, Cd, As, Hg) enter the environment from both natural sources and human activities, such as, e.g., industrial waste disposal, excessive and continuous use of pesticides and chemical fertilizers, mining/extraction activities and the dumping of household waste. All these activities cause accumulation in the soil, environment, and plants, entering the food chain and harming living organisms. Among various agricultural products, rice is particularly prone to accumulating heavy metals, which can cause serious health issues in humans [3]. Prolonged exposure to potentially toxic elements (PTEs) such as lead (Pb), arsenic (As), nickel (Ni), and cadmium (Cd) has been linked to an increased risk of malignant brain tumors, cardiovascular and kidney system disorders, as well as cognitive impairments in children [4]. Globalization has significantly contributed to soil and water pollution and contamination of harvested crops [5,6]. Since ubiquitous PTEs are chemically stable and non-biodegradable, humans are highly at risk of unavoidable exposure to these contaminants via food chains [7,8,9,10]. In addition to regional contamination, global climate change has been reported to influence the mobility and bioavailability of potentially toxic elements (PTEs) in agricultural soils, thereby increasing health risks associated with contaminated food crops in various regions worldwide [11]. Their findings reinforce the need for region-specific monitoring and health risk assessment of heavy metals in staple crops such as rice.

The heavy metals are classified by the International Agency for Research on Cancer (IARC) as follows: cadmium is classified as a human carcinogen (Group 1), Pb is a possible human carcinogen (Group 2B), and its inorganic compounds are considered a probable human carcinogen (Group 2A), while iAs (inorganic arsenic) is classified as a Group 1 substance, meaning it is carcinogenic to humans [12]. Arsenic (As) is a naturally occurring metalloid widely distributed in the environment and cannot be completely eliminated from ecosystems or the food chain [13]. It occurs in three major forms: organic arsenic, inorganic arsenic (iAs), and arsine gas, each differing in toxicity. Among these, inorganic arsenic (iAs) is considered the most toxic species. The term total arsenic (tAs) refers to the sum of organic and inorganic arsenic concentrations. Globally, risk assessments of arsenic contamination in food and the environment primarily focus on iAs due to its higher toxicity and stronger association with adverse health effects. Acute exposure to iAs may result in symptoms such as nausea, vomiting, abdominal pain, diarrhea, bruising, numbness, muscle cramps, and, in extreme cases, death [14]. Long-term exposure has been linked to chronic respiratory disorders, including laryngitis, bronchial infections, and an increased risk of lung cancer.

The Ministry of Agriculture of the Republic of Indonesia and the National Standardization Agency have regulated the maximum limits of heavy metal contamination in rice through the Minister of Agriculture Regulation No. 53 of 2018 concerning the Safety and Quality of Fresh Plant-Based Food [15] and SNI 6128:2020 [16] on rice, with the maximum limits set at 100 µg/kg for Cd, 200 µg/kg for Pb, and 200 µg/kg for inorganic arsenic as regulated in CXS_193e [17]. The global food body, Codex Alimentarius, regulates heavy metal contamination in Standard 193-1995 [17], which outlines the General Standard for Contaminants and Toxins in Food and Feed. Recommendations from the Joint FAO/WHO Expert Committee on Food Additives (JECFA) in Codex Standard 193-1995 also set toxicity values for each type of heavy metal, with the Provisional Tolerable Monthly Intake (PTMI) for cadmium set at 25 µg/kg bw/day. For inorganic arsenic, the lower reference dose for increased lung cancer incidence (BMDL_0.5_) was determined from epidemiological studies with a value of 3.0 µg/kg bw/day, using various assumptions to estimate total exposure to inorganic arsenic from drinking water and food. The European Food Safety Authority (EFSA) identified three health effects of lead exposure that provide sufficient consistent evidence for risk analysis: (1) neurodevelopmental disruption in toddlers, measured by a decrease in IQ; (2) cardiovascular effects in adults, measured by an increase in systolic blood pressure; and (3) an increased incidence of chronic kidney disease in adults, measured by a decrease in Glomerular Filtration Rate (GFR) [18]. Exposure to lead is associated with a wide range of effects, including various neurodevelopmental effects, mortality (mainly due to cardiovascular diseases), impaired renal function, hypertension, impaired fertility and adverse pregnancy outcomes. For adults, the adverse effects associated with the blood lead concentrations, for which the weight of evidence is greatest and most consistent, is a lead-associated increase in systolic blood pressure [19].

One of the main food crop commodities in the world is rice (*Oryza sativa* L.). Rice is an important source of energy, vitamins, and essential nutrients and is a staple food for more than half of the world’s population, spread across Europe, America, and Asia [3]. Indonesia ranks as the fourth-largest rice consumer globally, with a total consumption reaching 35.3 million metric tons in 2023, which equals 125 kg per capita [20]. Rice is a crop with a strong capacity to absorb cadmium, making it crucial to understand the pattern of cadmium contamination in the rice supply chain through predictive methods and to prevent and control it promptly. Heavy metals, particularly cadmium (Cd), arsenic (As), and lead (Pb), are highly bioavailable and tend to accumulate in rice grains due to the plant’s efficient uptake mechanisms. These toxic elements can be readily absorbed from contaminated soil and water, where they bind to soil particles or dissolve in water and then enter the rice plant through the roots [21].

Indonesia is an agrarian country that economically depends on agriculture. As mentioned, rice is the staple food in Indonesia, with an annual consumption of approximately 30.90 million tons [20]. With the increasing anthropogenic activities, such as irrigation with wastewater, as well as the use of agricultural fertilizers, pesticides, and organic manure in farming, rice may become a primary source of PTEs in the diet, particularly Cd, Pb, and As. Research focusing on heavy metal contamination in rice and its associated risk assessment in Indonesia is still limited, with existing studies mostly focused on specific regions that do not represent the entire country. Given the high consumption rate and food safety concerns, PTEs content in rice is an important issue to study both in Indonesia and globally. The potential health risks from long-term rice consumption containing PTEs need to be addressed [22].

This study aims to assess the exposure to PTEs (Cd, Pb and As) from rice in Indonesia by examining (i) the levels of Cd, Pb, and As in rice in Indonesia; (ii) the effect of rice processing into cooked rice on the levels of Cd, Pb, and As; (iii) the levels of exposure to PTEs Cd, Pb, and As from rice by age groups 0–59 months, 5–12 years, 13–18 years, 19–55 years, >55 years; (iv) to assess the health risk due to exposure to Cd, Pb, and As through the consumption of rice and cooked rice in Indonesia by age groups 0–59 months, 5–12 years, 13–18 years, 19–55 years, >55 years.

## 2. Materials and Methods

### 2.1. Chemicals and Materials

Cadmium, Lead, and Arsenic are standard solutions from Merck (Darmstadt, Germany). Ultrapure water was generated from a water purification system (Direct-Q^®^ 5UV, Merck, Darmstadt, Germany). Suprapur 60% nitric acid (HNO_3_) and suprapur 30% hydrogen peroxide (H_2_O_2_) were purchased from Merck (Darmstadt, Germany). A Working standard solution was prepared by serial dilution of the multi-element calibration standard-2A solution. Tuning solution for ICP-MS containing 1 μg/mL of Ba, Bi, Ce, Co, In, Li, and U in 2.5% HNO_3_ 0.5% HCl (Inorganic Ventures, Christianburg, VA, USA). All glassware and TFM vessels were cleaned by soaking in a 20% (*v*/*v*) HNO_3_ reagent grade for at least 24 h and rinsed with deionized water.

### 2.2. Sample Collection and Sample Preparation

The samples for this study were taken from the 30 largest rice-producing provinces in Indonesia based on rice production data from 2022 provided by Statistics Indonesia (BPS) [20], namely East Java, West Java, Central Java, South Sulawesi, South Sumatra, Lampung, North Sumatra, Banten, Aceh, West Nusa Tenggara, West Sumatra, South Kalimantan, East Nusa Tenggara, Central Sulawesi, West Kalimantan, Special Region of Yogyakarta, Southeast Sulawesi, West Sulawesi, Central Kalimantan, Bengkulu, Jambi, North Sulawesi, Gorontalo, Kalimantan, Riau, Papua, Maluku, Bangka Belitung, and North Kalimantan. Rice samples were taken from two cities or districts, each province’s largest rice production centers. Rice samples were collected by the Agricultural Quality Control Officers and Plant Pest Control Officers in each province, with 1 kg of rice from each location. Large laboratory samples were subsampled using the quartering or Koning technique to reduce the sample size; then, the test samples were homogenized by grinding/blending.

The rice samples to be tested were processed from 10 rice samples from 10 randomly selected provinces using two different cooking methods: with a rice cooker and by steaming using a pot and an aluminum steamer. The Indonesian population commonly uses these rice cooking methods. The cooking process used bottled drinking water to avoid heavy metal contamination from the water used to cook the rice. The Cd, Pb, and As levels in the water were below the LoD, which were 0.001 mg/L. The cooked rice from the 10 rice samples was then tested for heavy metals using a microwave digestion system and ICP-MS. The changes in the Cd, Pb, and As levels in rice after cooking were used as the processing factors for exposure calculations.

### 2.3. Microwave Digestion System and ICP-MS Analysis

The testing method used in this study refers to AOAC 2015.01 Heavy Metals in Food Inductively Coupled Plasma–Mass Spectrometry [23]. A 0.25 g sample was weighed into a vessel of the microwave digestion system, and 4 mL of Suprapur HNO_3_ 65% and 1 mL of H_2_O_2_ 30% were added. The vessel was placed into a Milestone Ethos Up microwave digestion system, and the digestion conditions were set at ramp temperature of 180 °C for 20 min, with a hold time of 15 min. The contents of the vessel were poured into a 20 mL volumetric flask, the vessel was rinsed, and the volumetric flask was brought to volume with ultrapure water. The solution was then diluted four times.

The prepared sample solution was analyzed quantitatively by ICP-MS. The Cd, Pb, and As levels in rice were analyzed using a Thermo ICAP RQ ICP-MS with the following conditions and ICP-MS settings: ion identifiers for ^111^Cd, ^208^Pb, and ^75^As. RF power was set at 27 MHz, with a nebulizer gas flow rate of 0.9 L/min, auxiliary gas flow rate of 0.8 L/min, and coolant gas flow rate of 14.0 L/min, using the KED operation mode, with a QCell flow rate of 4.5 mL/min and a spray chamber temperature of 3 °C. The Pb test was performed using ICP-MS with the following Limits of Detection (LoD): Cd 0.024 mg/kg, Pb 0.021 mg/kg, and As 0.03 mg/kg, and the Limits of Quantification (LoQ): Cd 0.08 mg/kg, Pb 0.07 mg/kg, and As 0.1 mg/kg.

The concentration of inorganic arsenic (iAs) in rice samples was estimated using a linear regression model. The regression equation was developed based on data extracted from 13 paired observations of total arsenic (tAs) and inorganic arsenic (iAs) concentrations in rice reported in published studies from various Asian countries (Table 1).

### 2.4. Human Risk Assessment and Exposure to Toxic Metals

#### 2.4.1. Estimated Daily Intake (EDI)

The daily exposure values for Cd, Pb, and As were calculated using a deterministic approach. This deterministic approach was chosen for this study based on the FAO/WHO exposure assessment framework (2009) [31], due to the availability of average food consumption data and average body weight data by age group obtained from the Individual Food Consumption Survey, Ministry of Health of the Republic of Indonesia [22]. The exposure level calculation was conducted for the average Cd, Pb, and As concentrations; the maximum value and exposure levels were also calculated using the maximum regulatory limits for heavy metals in food in Indonesia [16] as the heavy metal concentration.

The exposure to Cd, Pb, and As was calculated by multiplying the concentration by the rice consumption data and then dividing by the body weight data. This heavy metal exposure calculation was performed for the age groups of infants (0–59 months), children (5–12 years), adolescents (13–18 years), adults (19–55 years), and those over 55 years old. Rice consumption and body weight data by age group were obtained from the SKMI 2014 [22]. The formula used for the exposure calculation, based on information from FAO/WHO [31], is as follows:

$$EDI=\frac{\sum \;rice\;consumption\left(kg/person/day\right)\times \sum \;C\left(µg/kg\right)\;}{body\;weight\;(kgbw/person)}$$

where *C* (µg/kg) is the concentration of the heavy metal in rice; the resulting exposure value is expressed in µg/kg bw/day.

The exposure to Cd, Pb, and As in cooked rice was calculated by multiplying the exposure value for rice by the processing factor (Cd −76.65%; Pb 1403%; Total As −34%), which is obtained from the change in Cd, Pb, and As concentrations in rice after it has been processed into cooked rice.

#### 2.4.2. Risk Characterization

The risk characterization for Cd is carried out by calculating the risk value by comparing the exposure value with the total daily intake (TDI) value. The TDI value is obtained by dividing the PTMI value of 30 from Codex Alimentarius Standard 193-1995 [17]. The formula for calculating the Cd risk value is as follows [32]:

$$\%\mathrm{risk}\;=\;\frac{EDI}{TDI}\;\times \;100\%$$

The risk characterization for Pb is carried out by calculating the Margin of Exposure (MOE) based on the BMDL_01_ for cardiovascular effects in adults (1.5 µg/kg bw/day), BMDL_10_ for kidney function impairment in adults (0.63 µg/kg bw/day), and BMDL_01_ for neurotoxicity in children (0.5 µg/kg bw/day). The risk value for inorganic As is calculated by determining the MOE based on the reference BMDL for a 0.5% increase in lung cancer incidence due to i-As exposure, with a BMDL_0.5_ of 3.0 µg/kg bw/day. The MOE calculation formula is as follows:

Margin of Exposure=BMDL/EDI

High lead exposure in infants and children increases the likelihood of intelligence decline. The estimation of Pb exposure through diet related to IQ reduction in children uses the combined output from the bilinear and Hill models, where every 30 μg of lead per day is associated with a 1-point decrease in IQ [19].

$$\;IQ\;decrease=\frac{EDI(µg/kgbw/day)\times body\;weight(kgbw)}{30\;\mu g/day}$$

### 2.5. Statistical Analyses

The data were analyzed using Microsoft Excel. The mean, maximum values and SD of each metal were calculated to describe the metal contents in the rice samples. The data 10 log-transformation was performed in order to reach normal distribution, and thus further proceed with parametric statistical tests. Statistical analysis using ANOVA was conducted to compare the Pb and Cd test results among the different provinces. Student’s t-tests compared the 10 log-transformed means of each metal in the soaked and rinsed grains. Differences between the treatment methods used to prepare the rice were considered significant at *p*-value < 0.05.

**Figure 1:**
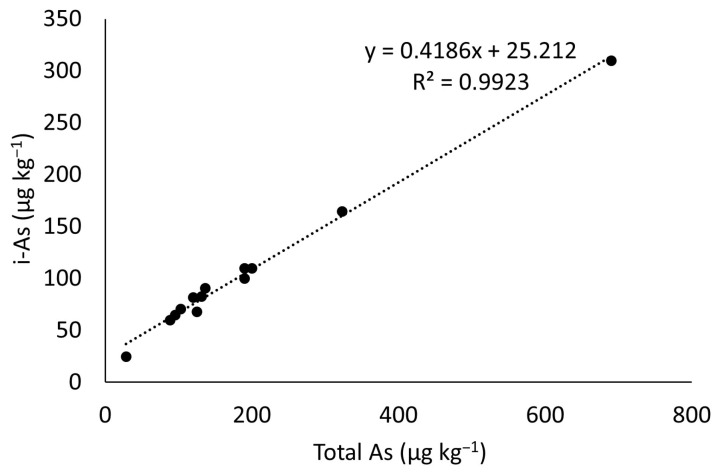
The regression equation between total Arsenic concentration and inorganic Arsenic concentration in rice, based on data extracted from 13 paired observations of total arsenic (tAs) and inorganic arsenic (iAs) concentrations in rice reported in published studies from various Asian countries [24,25,26,27,28,29,30], as presented in Table 1.

The relationship between t-As and iAs was analyzed using simple linear regression to derive a predictive equation. Statistical analysis was performed using Microsoft Excel, and the goodness-of-fit of the regression model was evaluated using the coefficient of determination (R^2^). A significance level of *p* < 0.05 was considered for all statistical tests. The regression equation can be seen in Figure 1.

Although Microsoft Excel was used for statistical analysis and regression modeling, the risk characterization was conducted manually. This step followed established health risk assessment guidelines using standard equations and reference values. No statistical software was employed for the risk characterization calculations.

## 3. Results and Discussion

### 3.1. Contamination of Rice with Cd, Pb, Total As, and Inorganic As

**Figure 2:**
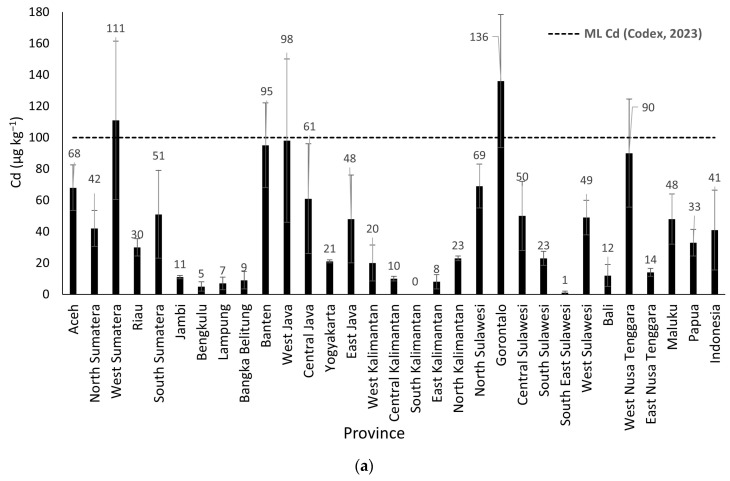

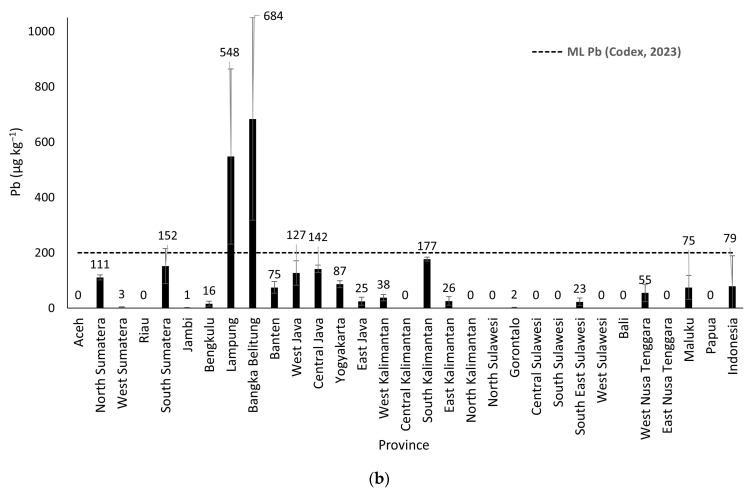
Average concentrations of Cd (**a**) and Pb (**b**) in rice from 30 provinces in Indonesia [17].

The concentrations of cadmium (Cd) and lead (Pb) in rice samples from 30 provinces are presented in Table 2. The average Cd level was 41 µg/kg, with values ranging from <LoD to 136 µg/kg. Notably, samples from West Sumatra (111 µg/kg) and Gorontalo (136 µg/kg) exceeded the national maximum limit for Cd in rice (100 µg/kg [15]), as illustrated in Figure 2. Higher Cd concentrations observed in certain samples may be attributed to anthropogenic sources, particularly the intensive use of chemical fertilizers and pesticides in rice production areas. The non-compliance with Good Agricultural Practices (GAP) could contribute to the elevated Cd levels. Phosphate fertilizers, while enhancing crop yield, are also known to contain cadmium as a contaminant, which can accumulate in rice and pose health risks [33].

The average Pb level was 79 µg/kg, with concentrations ranging from <LoD to 684 µg/kg. Two rice samples exceeded the maximum limit set by Codex Alimentarius (2023) [17], which is 200 µg/kg: Lampung (548 µg/kg) and Bangka Belitung (684 µg/kg). A single-factor ANOVA analysis showed a statistically significant difference in Pb and Cd concentrations across the provinces (*p*-value < 0.05). The elevated Pb level in rice from Lampung Province may be linked to industrial activities along the Way Sekampung River, which flows through several districts, including Tanggamus, East Lampung, and Central Lampung. This river system serves as a major irrigation source and passes through areas with high industrial density, where industrial waste discharge into rivers is a common problem. Such discharges can contaminate agricultural soils with heavy metals such as Pb and Cu, which subsequently accumulate in rice crops. In Bangka Belitung, the high Pb content in rice is likely due to contamination from mining activities, as West Bangka and South Bangka are regions with intensive mining operations. Heavy metals from mining can leach into both soil and water, affecting agricultural fields. These findings indicate that environmental contamination from industrial and mining activities plays a significant role in Pb accumulation in rice, particularly in provinces with known sources of pollution.

In this study, the heavy metal testing for As in rice was conducted using the tAs test, with an average value of tAs of 106 µg/kg, and minimum and maximum values ranging from 0 to 609 µg/kg. According to the Ministry of Indonesia Regulation No. 53 of 2018 [15] and SNI 6128:2020 [16] the maximum contamination limit for As has not been established. Codex Alimentarius, in CXS 193-1995 [17], sets the maximum contamination limit for i-As in rice at 200 µg/kg. The data for i-As was obtained through calculation using a regression equation based on the average i-As data and total As data in rice from Asia, which was derived from a literature study.

**Figure 3:**
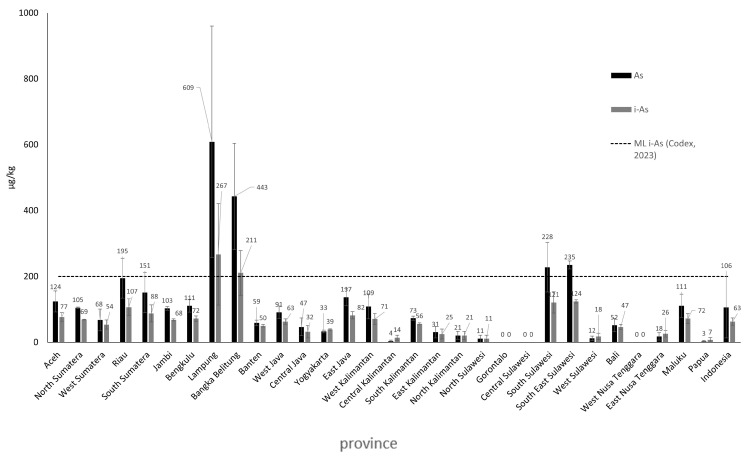
Average concentrations of total As and iAs in rice from 30 provinces in Indonesia [17].

The average concentration of inorganic arsenic (iAs) in rice samples from 30 provinces was 63 µg/kg, with values ranging from <LoD to 267 µg/kg. Two provinces reported iAs levels that exceeded the maximum limit set by Codex Alimentarius (2023) [17]: Lampung (267 µg/kg) and Bangka Belitung (211 µg/kg), as shown in Figure 3. A single-factor ANOVA analysis confirmed that both total arsenic (tAs) and iAs concentrations differed significantly across provinces (*p*-value < 0.05).

The high levels of i-As detected in rice samples from Lampung and Bangka Belitung are likely influenced by arsenic-contaminated irrigation water and mining activities in those areas. Mining operations can increase the arsenic concentration in surrounding agricultural soils through both dry deposition (airborne arsenic particles) and wet deposition (arsenic dissolved in rainwater), leading to elevated uptake by rice plants [34,35]. A study investigated arsenic and mercury concentrations in water and sediments from abandoned tin mining ponds (“air kolong”) in Bangka Belitung Province [36]. The results showed that arsenic concentrations in both water and sediments at several locations exceeded environmental quality standards, suggesting a potential source of contamination that could affect surrounding rice fields.

The maximum limits for heavy metal contamination in rice in Indonesia are regulated by the Ministry of Agriculture, Regulation No. 53 of 2018 [15], and Indonesian National Standard 6128:2020 [16]. The maximum limits for Cd, Pb, and inorganic Arsenic contamination in rice worldwide are regulated by the Codex Alimentarius [17]. The results of testing for Cd, Pb, and As in rice samples from Indonesia and the maximum contamination limits for Cd, Pb, and As are shown in Table 3.

### 3.2. Changes in Cd, Pb, and As Levels in Cooked Rice

The changes in Cd, Pb, and total As concentrations in cooked rice were determined by analyzing rice that had been prepared from previously tested raw rice samples. A total of 10 rice samples from 10 provinces with the highest Cd levels were selected for cooking using two different methods: electric rice cooker and steaming with commercial bottled drinking water (which had been pre-tested for Cd, Pb, and As to avoid contamination bias and confirmed to be below the LoD). The variations in Cd, Pb, and total As concentrations before and after cooking are presented in Table 4.

The average Cd concentration in the 10 selected raw rice samples was 139 µg/kg. After cooking, the mean Cd level in rice prepared using a rice cooker was 27 µg/kg, while that in steamed rice was 38 µg/kg. A t-test analysis showed no significant difference in Cd concentrations between the two cooking methods (*p*-value > 0.05). Therefore, the overall average Cd concentration in cooked rice was calculated as 34.5 µg/kg This indicates a 76.65% reduction in Cd concentration from raw to cooked rice, as represented by the processing factor.

The reduction in Cd levels can be attributed to the washing step during rice preparation. During washing, Cd is likely dissolved and removed, as it is commonly bound to proteins in the form of soluble amino acids and can also exist as water-soluble salts. According to Perelló et al. [37], the decline in Cd during cooking may be linked to its dissolution in soaking or cooking water, due to Cd’s affinity for proteins and its presence in water-soluble forms. Heating may enhance protein degradation, leading to the release of Cd as free salts, dissolved amino acids, or protein-bound forms into the water. This finding aligns with the results of Liu et al. [38], who reported that washing significantly reduces Cd levels, while cooking reduces Cd bioaccessibility. This is also consistent with the studies conducted by Hajeb et al. [39], Sharafi et al. [40], and Hadian et al. [1], which found that PTEs dissolve in water during the cooking process. According to Al-Saleh et al. [41], the findings clearly indicated that both soaking and rinsing grains in water are effective methods for decreasing cadmium content. In this study, since the cooking water was not discarded, the observed decrease in Cd concentration occurred primarily during the washing process.

The mean Pb concentration in the 10 selected raw rice samples was 52 ± 22 µg/kg. After cooking, the mean Pb concentration in rice prepared using a rice cooker was 737 ± 98 µg/kg, while that in steamed rice was 827 ± 111 µg/kg. A t-test analysis indicated that there was no significant difference in Pb concentrations between the two cooking methods (*p*-value > 0.05). Therefore, the overall average Pb concentration in cooked rice was calculated as 781.5 µg/kg. This represents a 14.03-fold increase in Pb concentration from raw rice to cooked rice, indicating that a significant Pb contamination occurred during the cooking process.

The increase in Pb concentration in cooked rice is likely due to contamination from cooking utensils. This observation is consistent with the findings of Ojezele et al. [42], who reported that heavy metal contamination in food can occur as a result of metal leaching from cookware. In their study, Pb levels in rice cooked using a glass beaker (as the control) were 0.01 ± 0.01 mg/kg, while rice prepared with various types of cookware showed Pb concentrations ranging from 0.035 ± 0.05 mg/kg to 3.22 ± 0.25 mg/kg. These results indicate that the use of different cooking vessels can significantly contribute to Pb contamination in food, most likely due to heavy metal leaching into the rice during the cooking process. The leaching of lead ions from cooking utensils into processed food was also reported in a study conducted by Tesfaw et al. [43]. Weidenhamer et al. [44] concluded that lead, aluminum, and cadmium can migrate from aluminum cookware during the cooking process and enter food at levels exceeding the recommended public health guidelines.

**Figure 4:**
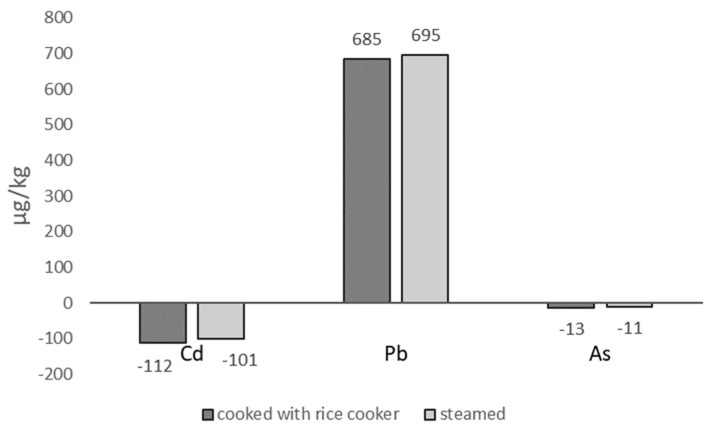
Changes in Cd, Pb, and As levels in rice processed with a rice cooker or steamed compared to raw rice.

The average total arsenic (As) concentration in the 10 selected rice samples was 33.5 ± 10 µg/kg, while the mean concentrations in cooked rice were 21.2 ± 12 µg/kg for rice cooker preparation and 23 ± 12 µg/kg for steaming. Based on one-way ANOVA (single factor), there were no statistically significant differences in total As concentrations between raw rice and cooked rice prepared by either method. These results are consistent with findings by Liao et al. [45], who reported a 2.6–24.4% reduction in total As content when rice was cooked under pressure using deionized water, compared to raw rice. Their study also found no significant differences in total As content between conventional and pressure cooking methods (*p* > 0.05). Similarly, Zhuang et al. [46] noted that cooking rice with a low water volume does not significantly reduce As concentrations. Several other studies also emphasize that washing rice before cooking can significantly reduce total As content in the final product [38,47]. Changes in Cd, Pb, and As levels in rice and cooked rice, whether processed with a rice cooker or steamed, can be seen in Figure 4.

The concentrations of Cd, Pb, and inorganic As (iAs) in cooked rice used for exposure assessment were estimated by multiplying the concentrations of these elements in raw rice by their respective processing factors. The calculated concentrations of Cd, Pb, and iAs used for exposure assessment are presented in Table 5. All values of Cd, Pb, total As, and iAsused in the exposure assessment are expressed on a wet weight basis.

### 3.3. Estimated Dietary Exposure

The order of Estimated Daily Intake (EDI) values for the potential toxic elements (PTEs) Cd, Pb, and As was as follows: children > adults. For both age groups, the order of PTEs was Pb > As > Cd. The estimated daily intake (EDI) values for all assessed metals followed a consistent trend, ranking as children > adolescents > adults > elderly. Despite having the lowest daily consumption (DC), children exhibited the highest EDI values for heavy metals, primarily due to their lower body weight (BW). Although elderly individuals generally have lower BW compared to adults, their EDI values remained lower than those of adults as a result of reduced daily consumption levels. These findings are consistent with the studies conducted by Hadian et al. [1] and Kukusamude et al. [3].

#### 3.3.1. Exposure Study of Cd in Raw and Cooked Rice

Estimated exposure to Cd from raw rice and cooked rice was calculated by multiplying rice consumption data by Cd concentration, and then dividing the result by body weight for each age group, as reported in the Indonesian Total Diet Study (TDS) in 2015 [22]. The average rice consumption and body weight for each age group are presented in Table 6.

A comparative summary of average Cd exposure from raw rice and cooked rice in this study versus exposure from cereal-based food groups in TDS is presented in Table 7. The estimated Cd exposure is categorized into six age groups: 0–59 months, 5–12 years, 13–18 years, 19–55 years, >55 years, and all ages. The average estimated Cd exposure from cooked rice across age groups and the general population ranged from 0.058 to 0.194; 0.050 to 0.167; 0.035 to 0.117; 0.032 to 0.108; 0.031 to 0.105; and 0.033 to 0.113 μg/kg BW/day, based on both mean and maximum values, respectively.

The highest Cd exposure was observed in the 0–59 months age group, while the lowest exposure occurred in individuals aged >55 years, for both raw and cooked rice. These results can be attributed to a higher ratio of body weight increase compared to the increment in rice consumption across age groups. The lowest exposure levels were observed in the 19–55 years and >55 years groups, while the highest exposure was consistently found in children under five years.

The average dietary exposure to Cd from raw rice in each age group and for the overall population in Indonesia, within the range of mean and maximum values, was 0.248–0.827; 0.214–0.713; 0.150–0.500; 0.138–0.459; 0.134–0.448; and 0.144–0.479 μg/kg bw/day, respectively.

According to the 2014–2015 Total Diet Study (TDS) conducted by the Indonesian Ministry of Health, in the Chemical Contaminants in Food Analysis [22], the estimated average Cd exposure for the Indonesian population ranged between 0.1262 and 0.3795 μg/kg bw/day. Among food groups, cereals and their products, including rice and cooked rice, contributed the most to Cd exposure, accounting for 68.27% of the total. Cd exposure values from raw rice in this study for each age group fell within the exposure range reported for cereal products in the TDS data. In contrast, Cd exposure values from cooked rice were lower than the range reported in TDS.

#### 3.3.2. Exposure Study of Pb in Raw and Cooked Rice

The estimated exposure to lead (Pb) from rice and cooked rice was calculated by multiplying rice consumption data by Pb concentration data, then dividing by body weight. As shown in Table 8, Pb exposure estimates are categorized into six age groups: 0–59 months, 5–12 years, 13–18 years, 19–55 years, >55 years, and all ages. The mean Pb exposure from rice across all age groups and the average consumption level in Indonesia ranged between 0.475 and 4.143; 0.410 and 3.574; 0.287 and 2.505; 0.264 and 2.302; 0.258 and 2.245; and 0.276 and 2.403 µg/kg bw/day, respectively.

At the same time, the mean Pb exposure from cooked rice across all age groups and average consumption levels ranged between 6.666 and 58.123; 5.751–50.152; 4.031–35.149; 3.704–32.297; 3.613–31.502; and 3.866–33.710 µg/kg bw/day, respectively. The lowest exposure was observed in the 19–55 years and >55 years age groups, while the highest exposure occurred in children aged 0–59 months. This is attributed to the higher ratio of body weight increase compared to the increase in rice consumption. The estimated dietary exposure to lead (Pb) from rice and cooked rice in the present study was found to be higher across all age groups compared to the Pb exposure levels reported for cereals and cereal-based products in the Indonesian Total Diet Study. This elevated exposure is primarily attributed to the higher concentrations of Pb detected in rice and its cooked form.

#### 3.3.3. Exposure Study of Total As and Inorganic As in Rice and Cooked Rice

The estimated exposure to inorganic As from rice and cooked rice was calculated by multiplying rice consumption data by the concentration of inorganic As, then dividing by body weight. The estimated As exposure values presented in Table 9 are divided into six age groups: 0–59 months, 5–12 years, 13–18 years, 19–55 years, >55 years, and all ages.

The average dietary exposure to inorganic As from rice in each age group and among the general population in Indonesia, within the range of mean and maximum values, was 0.435–1.686; 0.375–1.455; 0.263–1.020; 0.242–0.937; 0.236–0.914; and 0.252–0.978 μg/kg bw/day, respectively. Meanwhile, the average dietary exposure to inorganic As from cooked rice across the same age groups ranged from 0.348 to 1.171; 0.300 to 1.011; 0.210 to 0.708; 0.193 to 0.651; 0.189 to 0.635; and 0.202 to 0.679 μg/kg bw/day, respectively. These results are attributed to a higher body weight gain ratio relative to the increase in rice consumption. The lowest exposure was found in the 19–55 years and >55 years age groups, while the highest exposure occurred among toddlers (0–59 months).

The lowest and highest exposures for As are similar to those for Cd and Pb, where the lowest exposure is found in the 19–55 years and over 55 years age groups, while the highest exposure occurs in the toddler group (0–59 months) due to the higher weight gain ratio compared to the increase in rice consumption. The order of Estimated Daily Intake (EDI) values for As was as follows: children > adults. These findings are consistent with the studies conducted by Hadian et al. [1] and Kukusamude et al. [3]. The As exposure from rice and cooked rice in this study shows that the exposure in each age group is lower than the As exposure in the TDS data, both in terms of the average and maximum values.

### 3.4. Risk Characterization for Cd, Pb, and Inorganic As in Rice and Cooked Rice

The risk characterization of heavy metal exposure to Cd was conducted by comparing the exposure value with the Provisional Tolerable Monthly Intake (PTMI) for cadmium. According to Codex Alimentarius (2023) [17], the PTMI for cadmium is 25 μg/kg bw/day. The risk values for Cd exposure from rice and cooked rice are presented in Table 10. The infant age group (0–59 months) showed the highest risk value for Cd exposure from rice and cooked rice. The average risk value for cadmium exposure from rice and cooked rice across all age groups was low (<100% PTMI). When compared to Cd exposure for the total consumer and adults in Indonesia (TDS), the PTMI percentage values from rice and cooked rice are lower, with the % PTMI value for TDS being 168% PTMI for toddlers and 130% PTMI for school-aged children.

Referring to the BMDL_0.5_ threshold (3 µg/kg bw/day) for lung cancer associated with iAs exposure [19], the Margin of Exposure (MOE) values for rice across all age groups ranged from 6.90 ± 0.92 (0–59 months) to 12.41 ± 1.65 (>55 years), while for cooked rice, MOE values ranged from 8.62 ± 0.62 (children under five) to 15.51 ± 1.11 (>55 years), as presented in Table 11.

The MOE values for As exposure from both rice and cooked rice, based on the BMDL_0.5_ of 3 µg/kg bw/day, were <10,000, indicating a high level of health risk. According to EFSA [48], a minimum MOE value of 10,000 is considered necessary for substances that are both carcinogenic and genotoxic.

Risk characterization of lead (Pb) exposure was conducted by calculating the Margin of Exposure (MOE), comparing the estimated exposure levels to the Benchmark Dose Lower Confidence Limit (BMDL) values. According to EFSA [49], the BMDL_01_ representing the lower confidence limit of the benchmark dose associated with a 1% additional risk at the 95th percentile for neurotoxicity in children is 0.50 μg/kg bw/day. The same opinion also identified cardiovascular effects and nephrotoxicity in adults as potential critical adverse health outcomes of lead exposure, with BMDL_01_ and BMDL_10_ values of 1.50 and 0.63 μg/kg bw/day, respectively. The MOE values for Pb exposure in rice and cooked rice are provided in Table 12.

The MOE values based on the BMDL_01_ (1.5 µg/kg bw/day) for cardiovascular effects in adults, derived from the average Pb exposure from rice, were 5.68 ± 1.52 for the 19–55 years age group and 5.82 ± 1.56 for those aged >55 years. For cooked rice, the MOE values for the 19–55 years and >55 years age groups were 0.40 ± 0.11 and 0.42 ± 0.11, respectively.

The MOE values based on the BMDL_10_ (0.63 µg/kg bw/day) for kidney dysfunction in adults from rice consumption were 2.39 ± 0.64 for both the 19–55 years and >55 years age groups. For cooked rice, the MOE values in the 19–55 years and >55 years age groups were both 0.17 ± 0.05. The MOE values based on the BMDL_01_ (0.5 µg/kg bw/day) for Pb-related neurotoxicity in children were 1.05 ± 0.28 for the 0–59 months age group and 1.22 ± 0.33 for the 5–12 years group when exposed through rice. For cooked rice, MOE values were 0.08 ± 0.02 for the 0–59 months age group and 0.09 ± 0.02 for the 5–12 years group. According to the EFSA CONTAM Panel [18], MOE value of 10 or higher indicates a very low, if not negligible, risk of a significant increase in cases of chronic kidney disease, cardiovascular effects, or neurotoxicity in children. Furthermore, even an MOE above 1 is considered to represent a very low risk.

Based on the MOE calculations presented in Table 12, it can be observed that for rice, the MOE values related to cardiovascular effects and chronic kidney disease from average Pb exposure are above 1, indicating that the associated risk may exist but remains low. However, the MOE for neurotoxicity in children aged 0–59 months is below 1, suggesting a high level of risk. In contrast, all MOE values for cooked rice are below 1, indicating a high potential health risk across all endpoints.

The MOE value for Pb in this study was above 1.0, indicating a potential but relatively low health risk. For inorganic As, the MOE was found to be below 10,000, suggesting a possible health concern for consumers who regularly consume rice from the studied regions. When compared with international data, the estimated risks fall within a similar range to those reported in several other Asian countries. For instance, a study conducted in China reported that the target hazard quotient (THQ) values for Pb exceeded 1.0 for both adults and children, suggesting that rice consumption may pose a potential health risk [50]. Moreover, the MOE values in several major rice-producing and rice-consuming countries, such as China, Japan, Thailand, Bangladesh, and the United States, were also reported to be below 100. This indicates that the carcinogenic risks associated with inorganic arsenic (iAs) intake from rice require serious attention, and control measures should be implemented to reduce arsenic exposure through rice consumption [51]. Furthermore, carcinogenic risks of iAs exposure from white rice ingestion in China, Korea, Thailand, Vietnam, Bangladesh, and Cambodia were found to be at an unacceptable level (more than 1 case per 10,000 people) [52].

In this study, the Margin of Exposure (MOE) was estimated under a worst-case scenario, assuming complete absorption of heavy metals from rice. This conservative approach has inherent limitations, as it does not account for bioaccessibility and bioavailability, which may, in reality, reduce the fraction of metals absorbed systemically.

### 3.5. Impact of Pb Exposure on IQ Decline

High lead exposure in infants and children increases the likelihood of declining IQ. Estimating Pb exposure through diet related to IQ decline in children uses the combined outputs of bilinear and Hill models, where each 30 μg of Pb per day is associated with a 1-point decline in IQ [19]. The calculation of IQ decline due to Pb exposure was carried out for the 0–59 months age group (average body weight 11.67 kg) and the 5–12 years age group (average body weight 27.5 kg).

The estimated IQ reduction in toddlers and children based on the average and regulatory maximum exposure levels is presented in Table 13, indicating a high likelihood of IQ decline in both age groups due to Pb exposure from rice and cooked rice. The IQ decrement in toddlers (0–59 months) resulting from Pb exposure through rice was 0.18 ± 0.07 points, while in children (5–12 years), it was 0.38 ± 0.14 points. These reductions in IQ are lower than those estimated under regulatory maximum exposure scenarios. The IQ reduction due to Pb exposure from cooked rice was estimated at 2.59 ± 0.95 points for toddlers and 5.27 ± 1.93 points for children. According to Reuben et al. [53], childhood exposure to Pb has long-term consequences, as it is associated with lower cognitive function and socioeconomic status at the age of 38, as well as decreased IQ and reduced social mobility.

Children are typically the most vulnerable group, as exposure to lead from this source can lead to neurological impairments, hearing problems, stunted growth, and various other health issues. The societal impact, including diminished educational outcomes, lower IQ levels, and possibly higher crime rates, far exceeds any extra expenses that might be required to implement stricter regulations in cookware manufacturing to eliminate the use of lead and other toxic metals [54].

### 3.6. Study Limitations

This study has several limitations that should be acknowledged. First, dietary intake data were obtained from secondary sources, specifically the Indonesian Individual Food Consumption Survey (SKMI) [22], which may not fully reflect actual consumption patterns. Second, the rice samples analyzed were limited in number and collected from only two districts in each of the 30 provinces, which may not represent the actual rice consumed by the individuals surveyed in SKMI. Third, the cooking process was conducted using only two types of cooking utensils without replication, and the data used were based on conversion values rather than direct measurements. Fourth, the inorganic arsenic (iAs) concentration was not directly measured, but instead estimated by converting total arsenic (tAs) values using a regression model derived from literature-based data. Fifth, the exposure assessment for heavy metals in cooked rice was estimated by multiplying the heavy metal content in raw rice by a processing factor, which was calculated from only ten rice samples that were cooked. Sixth, in this study, risk characterization was conducted under a worst-case scenario, assuming complete absorption of heavy metals from rice. This conservative approach has inherent limitations, as it does not account for bioaccessibility and bioavailability, which may, in reality, reduce the fraction of metals absorbed systemically. Therefore, the overall risk estimates presented in this study may represent an overestimation of actual exposure. Consequently, the true health risks associated with rice consumption are likely lower than those indicated by the current assessment. These limitations highlight the need for future studies to expand sampling coverage, incorporate a greater diversity of cooking equipment, and directly analyze inorganic arsenic levels in order to produce more comprehensive and representative results.

## 4. Conclusions

In this study, the average levels of heavy metals Cd, Pb, and As in rice in Indonesia are below the maximum contamination limits set by Codex Alimentarius. The average Cd level was 41 µg/kg, with values ranging from <LoD to 136 µg/kg. The average Pb level was 79 µg/kg, with concentrations ranging from <LoD to 684 µg/kg. In this study, the heavy metal testing for As in rice was conducted using the tAs test, with an average value of tAs of 106 µg/kg, and minimum and maximum values ranging from <LoD to 609 µg/kg. The estimated average concentration of inorganic arsenic (iAs) in rice samples from 30 provinces was 63 µg/kg, with values ranging from <LoD to 267 µg/kg. The concentrations of heavy metals in rice in Indonesia are in the following order: Pb > iAs > Cd. The Cd levels in rice from West Sumatra and Gorontalo, as well as Pb and As levels in rice from Lampung and Bangka Belitung, exceed the Codex Alimentarius maximum contamination limits. High concentrations of Cd and Pb in rice may be caused by various anthropogenic activities, including the use of chemical fertilizers and pesticides. High levels of As may be linked to mining activities and anthropogenic pollution that contaminate soil and irrigation in rice fields.

The Cd levels in rice decreased by a factor of 0.76 compared to those in raw rice. This reduction occurs due to washing and heating during the rice cooking process. Pb levels in rice increased by a factor of 14.03 compared to the levels in raw rice, likely due to Pb leaching from cooking equipment used during rice preparation. The As levels in rice showed no significant decrease compared to those in raw rice, as the cooking process with low water volumes does not significantly reduce As content.

The average risk values for Cd exposure from raw and cooked rice for all age groups are considered low (<100% PTMI). The Margin of Exposure (MOE) values for Pb exposure, based on BMDL_01_ (1.5 μg/kg bw/day) for cardiovascular effects in adults, BMDL_10_ (0.63 μg/kg bw/day) for kidney function impairment in adults, and BMDL_01_ (0.5 μg/kg bw/day) for neurotoxicity in children, are all below 10,000, indicating a high risk. Similarly, the MOE for As exposure, based on BMDL_0.5_ for lung cancer (3 µg/kg bw/day) for raw and cooked rice, is also below 10,000, indicating a high risk. The estimated IQ reduction due to Pb exposure from rice for the age group of toddlers (0–59 months) from rice is 0.18 ± 0.07 points, while for children (5–12 years), it is 0.38 ± 0.14 points. These reductions are lower than the IQ decrease observed at the maximum regulatory exposure limits. However, the IQ reduction from Pb exposure in rice for toddlers is 2.59 ± 0.95 points, and for children, it is 5.27 ± 1.93 points.

The results highlight the critical importance of strengthening food safety risk management practices in both rice production and processing. However, these results should be interpreted with caution due to several limitations, including the reliance on secondary dietary data, limited sampling scope, indirect estimation methods for inorganic arsenic, and the use of a small number of cooking scenarios. Future studies should address these limitations by incorporating direct measurements, expanding geographic and sample coverage, and exploring a wider range of cooking practices to enhance the accuracy and generalizability of the findings.

## Figures and Tables

**Table 1:** Concentrations of total arsenic (tAs) and inorganic arsenic (iAs) in rice from various Asian countries.

Country	tAs (µg/kg)	iAs (µg/kg)	Reference
Thailand	125	68.3	Ruangwises et al., 2012 [24]
Iran	120	82	Cano-Lamadrid et al., 2015 [25]
Bangladesh	131	83	Chen et al., 2016 [26]
Japan	190	100	Lee et al., 2018 [27]
South Korea	88	60	Lee et al., 2018 [27]
Malaysia	103	71	Chanpiwat et al., 2019 [28]
Turki	323	165	Chanpiwat et al., 2019 [28]
Iraq	95	65	Chanpiwat et al., 2019 [28]
Vietnam	136	91	Chanpiwat et al., 2019 [28]
Taiwan	200	110	Rahman et al., 2011 [29]
India	28	25	Chanpiwat et al., 2019 [28]
Taiwan	190	110	Shakoor, 2019 [30]
Bangladesh	690	310	Rahman et al., 2011 [29]

**Table 2:** Cd, Pb, and total As levels in rice from 30 provinces in Indonesia.

Provinces	Cd Level	Pb Level	Total As Level
	Dry Basis (µg/kg)	Dry Basis (µg/kg)	Dry Basis (µg/kg)
Aceh	68 ± 29	<LoD	124 ± 64
North Sumatera	42 ± 23	111 ± 19	105 ± 5
West Sumatera	111 ± 101	<LoD	68 ± 66
Riau	30 ± 11	<LoD	195 ± 121
South Sumatera	51 ± 56	152 ± 127	151 ± 122
Jambi	<LoD	<LoD	103 ± 12
Bengkulu	<LoD	<LoD	111 ± 39
Lampung	<LoD	548 ± 633	609 ± 703
Bangka Belitung	<LoD	684 ± 732	443 ± 323
Banten	95 ± 54	75 ± 42	59 ± 17
West Java	98 ± 104	127 ± 88	91 ± 39
Central Java	61 ± 70	142 ± 27	47 ± 54
Special region of Yogyakarta	<LoD	87 ± 25	33 ± 8
East Java	48 ± 56	25 ± 29	137 ± 52
West Kalimantan	<LoD	38 ± 23	109 ± 76
Central Kalimantan	<LoD	<LoD	<LoD
South Kalimantan	<LoD	177 ± 14	73 ± 13
East Kalimantan	<LoD	26 ± 31	31 ± 36
North Kalimantan	<LoD	<LoD	<LoD
North Sulawesi	69 ± 28	<LoD	<LoD
Gorontalo	136 ± 85	<LoD	<LoD
Central Sulawesi	50 ± 44	<LoD	<LoD
South Sulawesi	<LoD	<LoD	228 ± 151
Southeast Sulawesi	<LoD	23 ± 27	235 ± 24
West Sulawesi	49 ± 22	<LoD	<LoD
Bali	<LoD	<LoD	52 ± 40
West Nusa Tenggara	90 ± 69	55 ± 63	<LoD
East Nusa Tenggara	<LoD	<LoD	<LoD
Maluku	48 ± 32	75 ± 87	111 ± 70
Papua	33 ± 17	<LoD	<LoD
INDONESIA	41 ± 51	79 ± 110	106 ± 93

LoD: Cd 24 µg/kg; Pb 21 µg/kg; total As 30 µg/kg.

**Table 3:** Heavy metal levels in the tested rice samples and maximum limits for heavy metals.

Heavy Metal Contamination in Rice	Cd (µg/kg)	Pb (µg/kg)	As (µg/kg)	iAs (µg/kg)
Mean ± SD	41 ± 51	79 ± 110	106 ± 93	63 ± 11
Minimum–maximum	<LoD–136	<LoD–684	<LoD–609	<LoD–267
Maximum limit				
Ministry of Agriculture of Indonesia (2018) [15]	100	200	-	-
Indonesian National Standard SNI 6128:2020 [16]	100	200	-	-
Codex Alimentarius (2023) [17]	400	200	-	200

**Table 4:** Changes in concentrations of Cd, Pb, and total As from raw rice to cooked rice (%).

Heavy Metal	Rice Dry Basis (µg/kg)	Cooked Rice Dry Basis (µg/kg)	Changes in Heavy Metal Levels (%)
Cd	139.1 ± 14.6	34.5 ± 10	−76.65
Pb	52 ± 22	781.5 ± 104.5	1403
Total As	33.5 ± 10	22.1 ± 12	−34

**Table 5:** Levels of Cd, Pb, and As according to regulations and analysis, for exposure assessment.

Maximum Limits/Heavy Metal Levels ^a^	Cd (µg/kg)	Pb (µg/kg)	As (µg/kg)	iAs (µg/kg)
Minister of Agriculture Regulation No. 53 of 2018 [15], SNI 6128:2020 [16]	100	200	-	-
Codex Alimentarius (2023) [17]	400	200	-	200
Results of the analysis				
Average concentration in raw rice	37 ± 6	71 ± 26	96 ± 22	65 ± 10
Maximum concentration in raw rice	123.5	619	541	252
Average concentration in cooked rice	8.6 ± 1.44	996 ± 365	63 ± 7.5	52 ± 4
Maximum concentration in cooked rice	29	8685	357	175

^a^ heavy metal concentration reported on the wet weight basis.

**Table 6:** Rice consumption and average body weight by age group according to TDS (2015) [22].

Age Group	Rice Consumption (g/d)	Average Body Weight (kg)
0–59 months	78.10	11.67
5–12 years	158.80	27.50
13–18 years	187.50	46.33
19–55 years	215.20	57.87
>55 years	189.70	52.30
All ages	197.10	50.78

**Table 7:** Average Cd exposure from rice and cooked rice in this study versus exposure from cereal-based food groups in the Total Diet Study (TDS) 2015.

Age Group	Cd Exposure (µg/kg bw/Day)					
	Raw Rice		Cooked Rice		Total Diet Study (TDS)	
	Mean ± SD	Max	Mean ± SD	Max	Average	Max
0–59 months	0.248 ± 0.040	0.827	0.058 ± 0.010	0.194	0.212	0.687
5–12 years	0.214 ± 0.035	0.713	0.050 ± 0.008	0.167	0.184	0.559
13–18 years	0.150 ± 0.024	0.500	0.035 ± 0.006	0.117	0.123	0.372
19–55 years	0.138 ± 0.022	0.459	0.032 ± 0.005	0.108	0.113	0.335
>55 years	0.134 ± 0.022	0.448	0.031 ± 0.005	0.105	0.111	0.327
All ages	0.144 ± 0.023	0.479	0.033 ± 0.006	0.113	0.126	0.380

**Table 8:** Average Pb exposure from rice and cooked rice in this study versus exposure from cereal-based food groups in the Total Diet Study (TDS) 2015.

Age Group	Pb Exposure (µg/kg bw/Day)					
	Rice		Cooked Rice		Total Diet Study (TDS)	
	Mean ± SD	Max	Mean ± SD	Max	Average	Max
0–59 months	0.475 ± 0.174	4.143	6.666 ± 2.443	58.123	1.032	3.470
5–12 years	0.410 ± 0.150	3.574	5.751 ± 2.108	50.152	0.693	2.621
13–18 years	0.287 ± 0.105	2.505	4.031 ± 1.477	35.149	0.444	1.702
19–55 years	0.264 ± 0.097	2.302	3.704 ± 1.357	32.297	0.422	1.515
>55 years	0.258 ± 0.094	2.245	3.613 ± 1.324	31.502	0.333	1.388
All ages	0.276 ± 0.101	2.403	3.866 ± 1.417	33.710	0.472	1.736

**Table 9:** Total As (tAs) and inorganic As (iAs) exposure in rice and cooked rice in this study versus tAs exposure based on the Total Diet Study (TDS) 2015 [22].

Age Group	tAs Exposure (µg/kg bw/Day)				iAs Exposure (µg/kg bw/Day)				tAs Exposure (µg/kg bw/Day)	
	Rice		Cooked Rice		Rice		Cooked Rice		Total Diet Study (TDS)	
	Mean ± SD	Max	Mean ± SD	Max	Mean ± SD	Max	Mean ± SD	Max	Average	Max
0–59 months	0.642 ± 0.147	3.621	0.422 ± 0.050	2.389	0.435 ± 0.067	1.686	0.348 ± 0.027	1.171	2.536	3.221
5–12 years	0.554 ± 0.127	3.124	0.364 ± 0.043	2.062	0.375 ± 0.058	1.455	0.300 ± 0.023	1.011	2.402	2.914
13–18 years	0.389 ± 0.089	2.189	0.255 ± 0.030	1.445	0.263 ± 0.040	1.020	0.210 ± 0.016	0.708	1.464	1.796
19–55 years	0.357 ± 0.082	2.012	0.234 ± 0.028	1.328	0.242 ± 0.037	0.937	0.193 ± 0.015	0.651	1.358	1.650
>55 years	0.348 ± 0.080	1.962	0.229 ± 0.027	1.295	0.236 ± 0.036	0.914	0.189 ± 0.015	0.635	1.279	1.564
All ages	0.373 ± 0.085	2.100	0.245 ± 0.029	1.386	0.252 ± 0.039	0.978	0.202 ± 0.016	0.679	1.529	1.868

**Table 10:** Risk value (%PTMI) for Cd exposure from rice and cooked rice in Indonesia.

Age Group	Cadmium Exposure Risk Value (%PTMI) (% PTMI)				
	Rice			Cooked Rice	
	Mean ± SD	Max	ML ^a^	Mean ± SD	Max
0–59 months	29.7 ± 4.8	99.18	80.31	6.9 ± 1.2	23.3
5–12 years	26.6 ± 4.2	85.58	69.29	6.0 ± 1.0	20.1
13–18 years	18.0 ± 2.9	59.98	48.56	4.2 ± 0.7	14.1
19–55 years	16.5 ± 2.7	55.11	44.62	3.8 ± 0.6	12.9
>55 years	16.1 ± 2.6	53.76	43.53	3.7 ± 0.6	12.6
All ages	17.2 ± 2.8	57.53	46.58	4.0 ± 0.7	13.5

^a^ ML: Maximum level of cadmium in rice based on Indonesian regulation [15].

**Table 11:** Risk characterization as the Margin of Exposure (MOE) value for inorganic arsenic exposure in rice and cooked rice.

Age Group	MOE ^a^				
	Rice			Cooked Rice	
	Mean ± SD	Max	ML ^b^	Mean ± SD	Max
0–59 months	6.90 ± 0.92	1.78	2.24	8.62 ± 0.62	2.56
5–12 years	7.99 ± 1.07	2.06	2.60	9.99 ± 0.71	2.97
13–18 years	11.40 ± 1.52	2.94	3.71	14.26 ± 1.02	4.24
19–55 years	12.41 ± 1.65	3.20	4.03	15.51 ± 1.11	4.61
>55 years	12.72 ± 1.70	3.28	4.14	15.91 ± 1.14	4.73
All ages	11.89 ± 1.59	3.07	3.86	14.86 ± 1.06	4.42

^a^ MOE based on BMDL_0.5_ of lung cancer 3 µg/kg bw/day; ^b^ ML: maximum level of inorganic arsenic based on CXS 193-1995 General Standard For Contaminants And Toxins In Food And Feed [17].

**Table 12:** Risk characterization as the Margin of Exposure (MOE) value for lead (Pb) exposure in rice and cooked rice.

Age Group	MOE				
	Rice			Cooked Rice	
	Mean ± SD	Max	ML ^a^	Mean ± SD	Max
BMDL_01_ for cardiovascular in adults					
19–55 years	5.68 ± 1.52	0.59	2.02	0.40 ± 0.11	0.40
>55 years	5.82 ± 1.56	0.60	2.07	0.42 ± 0.11	0.42
BMDL_10_ for nephrotoxicity effects in adults					
19–55 years	2.39 ± 0.64	0.25	0.85	0.17 ± 0.05	0.17
>55 years	2.45 ± 0.66	0.25	0.87	0.17 ± 0.05	0.17
BMDL_01_ for neurotoxicity in young children					
0–59 months	1.05 ± 0.28	0.11	0.37	0.08 ± 0.02	0.08
5–12 years	1.22 ± 0.33	0.13	0.43	0.09 ± 0.02	0.09

^a^ ML: Maximum level of lead in rice based on Indonesian regulation [15].

**Table 13:** Reduction in IQ scores in infants and children based on lead (Pb) exposure levels from rice and cooked rice.

Age Group	Pb Exposure (µg/kg)		Reduction in IQ	
	Rice	Cooked Rice	Rice	Cooked Rice
0–59 months				
Mean ± SD	0.48 ± 0.17	6.67 ± 2.44	0.18 ± 0.07	2.59 ± 0.95
Regulation ML ^a^	1.34	-	0.52	-
5–12 years				
Mean ± SD	0.41 ± 0.15	5.75 ± 2.11	0.38 ± 0.14	5.27 ± 1.93
Regulation ML ^a^	1.15	-	1.06	-

Pb exposure of 30 µg/day reduces IQ by 1 point [19]. ^a^ ML: Maximum level of lead in rice based on Indonesian regulation [15].

## Data Availability

The original contributions presented in this study are included in the article. Further inquiries can be directed to the corresponding authors.

## Abbreviations

The following abbreviations are used in this manuscript:

Al	Aluminum
As	Arsenic
Ba	Barium
BMDL	Benchmark Dose Lower Confidence Limit
BW	Body weight
Cd	Cadmium
Cr	Chromium
DC	Daily consumption
EDI	Estimated Daily Intake
EFSA	European Food Safety Authority
EU	European Union
FAO	Food and Agriculture Organization of the United Nations
GAP	Good Agricultural Practices
GFR	Glomerular Filtration Rate
Hg	Mercury
IARC	International Agency for Research on Cancer
i-As	Inorganic arsenic
ICP-MS	Inductively Coupled Plasma–Mass Spectrometry
IQ	Intelligence Quotient
JECFA	Joint Expert Committee on Food Additives
LoD	Limit of Detection
LoQ	Limit of Quantification
MOE	Margin of Exposure
Ni	Nickel
Pb	Lead
PTEs	Potentially toxic elements
PTMI	Provisional Tolerable Monthly Intake
SD	Standard deviation
SKMI	Indonesian Individual Food Consumption Survey
t-As	Total arsenic
TDI	Total Daily Intake
TDS	Total Diet Study
Ti	Titanium
WHO	World Health Organization
